# Atypical Mpox Presentation With Proctitis in a Puerto Rican Patient: A Case Report

**DOI:** 10.7759/cureus.99869

**Published:** 2025-12-22

**Authors:** Valeria E López Martínez, Melvin Arroyo Flores, Kimberly N Pagán Reyes, Fabián Mercado Nieves, Janiabeth Vega Maldonado

**Affiliations:** 1 Medical Education, Universidad Autónoma de Guadalajara School of Medicine, Guadalajara, MEX; 2 Family Medicine, Manati Medical Center (MMC), Manatí, PRI; 3 Medical Education, San Juan Bautista School of Medicine, Caguas, PRI; 4 Medical Education, Universidad Central del Caribe School of Medicine, Bayamón, PRI; 5 Infectious Disease, Manati Medical Center (MMC), Manatí, PRI

**Keywords:** hpv, hsv, monkeypox virus, mpox virus, orthopoxvirus, proctitis, puerto rico

## Abstract

Mpox, formerly known as Monkeypox, is a viral illness that has spread beyond its previous endemic areas and is now reported in the Caribbean. This report describes a 32-year-old man from Puerto Rico with a history of Herpes simplex virus and human papillomavirus who went to the emergency room with pelvic pain, fever, fatigue, rash, and constipation. Three days later, the rash turned into pustules. A CT scan showed rectal inflammation, and PCR testing confirmed Mpox infection. The patient had recently traveled to Minnesota and reported unprotected sexual contact with an unknown male partner approximately one week before his symptoms began. Initially, the presentation was attributed to bacterial proctitis, which delayed the correct diagnosis. After supportive care with antivirals and topical treatment, symptoms improved, and he was discharged with home isolation instructions. This case illustrates how Mpox can resemble other sexually transmitted infections and highlights the importance of including it in the differential diagnosis when evaluating patients in Puerto Rico, where it is still uncommon. Because atypical Mpox may present with limited lesions, minimal systemic symptoms, and early anorectal complaints, it can be mistaken for more common STIs, underscoring the need for clinicians in low-incidence regions to remain vigilant.

## Introduction

Monkeypox virus, now called Mpox, is a double-stranded DNA virus from the Orthopoxvirus genus in the Poxviridae family. It is a zoonotic infection that spreads mainly through close contact with infected individuals, contaminated objects, or animals [1]. The illness often begins with fever, fatigue, muscle aches, and swollen lymph nodes. This is followed by a rash that progresses through several stages, starting as flat macules, then raised papules, vesicles, and pustules that eventually scab over within two to three weeks. Lesions typically appear in the anogenital area, trunk, face, and limbs. It has been reported that approximately 14-36% of patients develop proctitis [1-3]. However, newer cases often involve rectal pain and predominantly localized lesions, which differ from the more extensive skin involvement reported in earlier outbreaks [4].

Because Mpox can present with localized or atypical rashes, it should be considered in the differential diagnosis of patients with sexually transmitted infections like skin lesions. In certain patients, Mpox may present with only a small number of deep, umbilicated pustules confined to the anogenital region rather than a disseminated rash. These localized lesions can closely resemble those seen in herpes simplex infections, early syphilis, or lymphogranuloma venereum, creating significant diagnostic overlap. As a result, distinguishing Mpox from more common sexually transmitted infections can be challenging in the early stages, particularly in settings where clinical suspicion remains low. Laboratory confirmation is typically achieved through serologic studies, lesion-based nucleic acid amplification tests, immunochemical techniques, or electron microscopy [5].

Recent outbreaks have shown that Mpox may not always follow its classic clinical pattern, especially in immunocompromised individuals. Instead of the widespread cutaneous involvement and prominent systemic symptoms, some patients develop only a few localized pustules or mild, nonspecific symptoms that can easily look like other sexually transmitted infections. Early detection can be challenging for this reason, especially in areas where Mpox is uncommon and not routinely considered. Recognizing these atypical patterns early can help clinicians avoid delays in diagnosis and consider Mpox when evaluating patients with STI-like symptoms.

Despite the global increase in cases, there is limited research describing the Mpox virus among individuals living in the Caribbean. Atypical manifestations in immunocompromised hosts may closely resemble other sexually transmitted conditions, contributing to misdiagnosis and delayed management [6]. This case highlights the importance of maintaining clinical awareness and prompt diagnostic testing in non-endemic regions such as Puerto Rico. We present this case to illustrate an atypical presentation of an Mpox infection and to underscore the need for early recognition and timely intervention to reduce transmission risk. 

## Case presentation

A 32-year-old Puerto Rican man with a past medical history of Herpes simplex virus and human papillomavirus diagnosed in 2023 presented to the ER with pelvic and abdominal pain, fever, chills, malaise, and constipation requiring straining. One week before admission, he developed low-grade fever and generalized fatigue, which he mistakenly attributed to a previously drained gluteal abscess with similar systemic symptoms. At a nearby Center for Diagnostic and Treatment (CDT), he was empirically started on ciprofloxacin and ketoconazole. Over the next several days, he reported an erythematous rash on the neck and upper chest that progressively spread to the elbows bilaterally. Subsequently, the patient noted that the initial erythematous rash had resolved, followed by the appearance of two pustular lesions on the neck. During the same period, he experienced rectal discomfort and painful defecation associated with diffuse perianal erythema and scant hematochezia, without visible pustules. His constipation gradually improved, but he continued to notice intermittent blood streaks in his stool.

On initial examination, he was afebrile, hemodynamically stable, and appeared in mild distress due to rectal pain. Scattered umbilicated pustules and crusted lesions were observed on the neck, trunk, extremities, and genital region. Perianal erythema was present without abscess or ulceration. 

**Figure 1:**
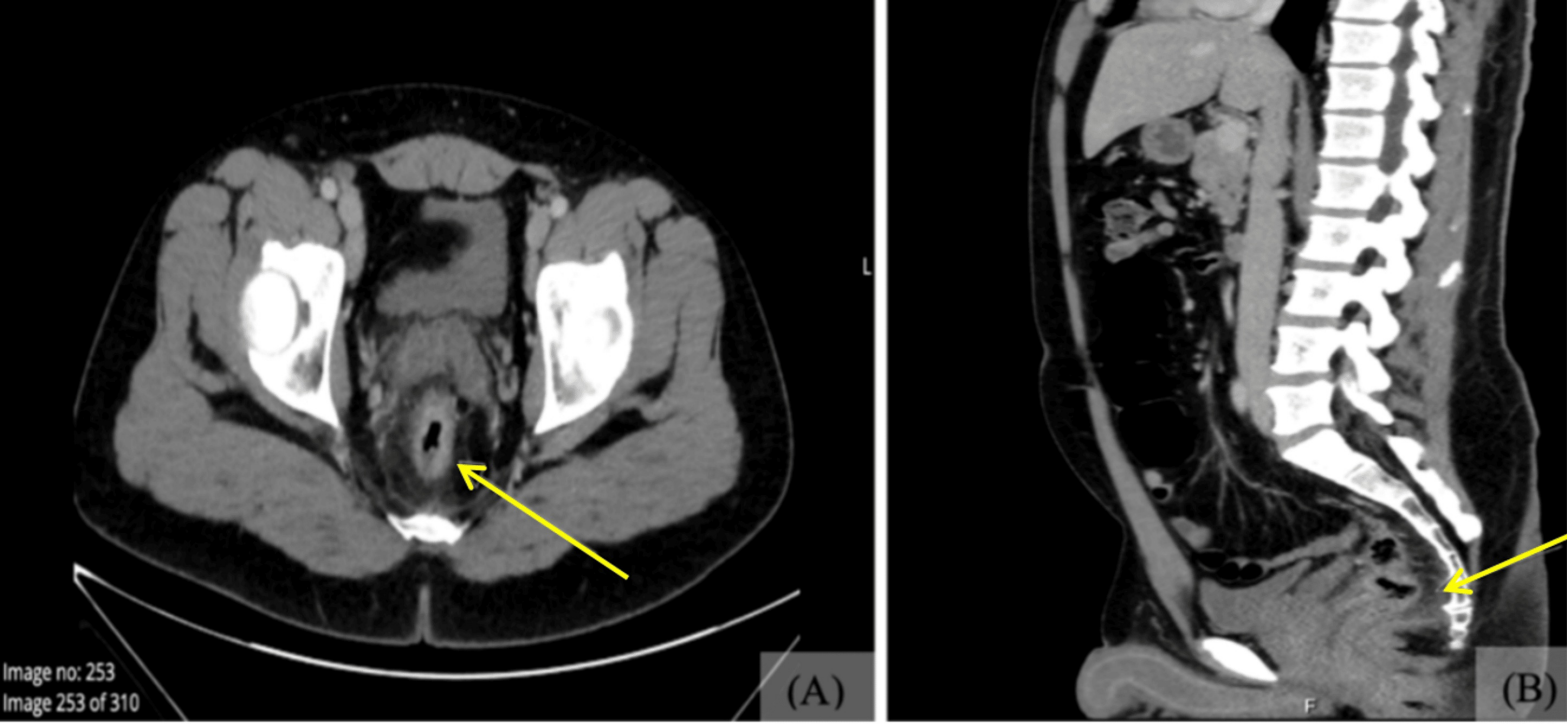
Contrast-enhanced CT of the abdomen and pelvis: (A) Axial view showing noticeable thickening and enhancement of the rectal wall (arrow). (B) Sagittal view demonstrating circumferential thickening and mucosal enhancement of the rectal wall (arrow), consistent with inflammatory changes.

The patient was admitted on October 11, 2025, for further evaluation of suspected proctitis. The admission was supported by a CT scan of the abdomen and pelvis with contrast, which revealed circumferential rectal wall thickening with mucosal hyperenhancement and fat stranding, compatible with inflammatory proctitis (Figure 1). Admission laboratory studies were notable for leukocytosis and elevated inflammatory markers, while renal function remained within normal limits (Table 1). The elevated CRP and leukocytosis further supported an active inflammatory process and aligned with the patient’s worsening anorectal symptoms. Initial management included Ciprofloxacin 400 mg IV every 12 hours and Metronidazole 500 mg IV every eight hours. Ciprofloxacin was initiated empirically due to concern for bacterial proctitis. Although the patient expressed concern that the rash might represent an allergic reaction to ciprofloxacin, the clinical team evaluated this possibility as part of the diagnostic reasoning process. Based on evolving clinical findings and laboratory results, the antimicrobial regimen was subsequently adjusted.

**Table 1:** Admission laboratory results Abnormal values are tagged as high or low to emphasize systemic inflammation. These abnormalities, although nonspecific, supported the presence of an active infectious or inflammatory process.

Laboratory Test	Patient Value	Reference Range
Complete Blood Count		
White blood cells (×10³/µL)	16.92 (H)	3.98–10.04
Red blood cells (×10⁶/µL)	4.88	4.63–6.08
Hemoglobin (g/dL)	14.5	13.7–17.5
Hematocrit (%)	42.5 (L)	43.5–53.7
MCV (fL)	87.1	79–94.8
MCH (pg)	29.7	25.6–32.2
MCHC (g/dL)	34.1	32.2–36.5
RDW (%)	12.3	11.6–14.4
Platelets (×10³/µL)	285	163–369
Neutrophils (%)	75	34.0-71.1
Lymphocytes (%)	14	19.3-53.1
Monocytes (%)	6	4.7-12.5
Bands (%)	1	5-10
Reactive lymphocytes (%)	3	0-10
Eosinophils (%)	1	0.7-7.0
Metabolic Panel/ Liver Function		
Sodium (mmol/L)	139	136–145
Potassium (mmol/L)	4.5	3.5–5.1
Chloride (mmol/L)	107	98–107
Bicarbonate (mmol/L)	26	21–32
BUN (mg/dL)	15	7–18
Creatinine (mg/dL)	1.02	0.55–1.30
Glucose (mg/dL)	105 (H)	70–99
Calcium (mg/dL)	9.1	8.5–10.1
Total bilirubin (mg/dL)	0.45	0.20–1.00
AST (IU/L)	52 (H)	15–37
ALT (IU/L)	81 (H)	13–61
Alkaline phosphatase (IU/L)	104	45–117
Total protein (g/dL)	8.0	6.4–8.2
Albumin (g/dL)	3.9	3.4–5.0
Globulin (g/dL)	4.1	2.0-3.5
C-reactive protein (mg/dL)	3.61 (H)	0.00–0.29
Urinalysis		
pH	5.5	5.0–8.0
Specific gravity	1.027	1.003–1.035
Protein	Negative	Negative
Glucose	Negative	Negative
Ketones	Negative	Negative
Blood	Negative	Negative
Leukocyte esterase	Negative	Negative
Nitrites	Negative	Negative
Urobilinogen (EU/dL)	0.2	0.2–1.0
WBC (urine) (cells/µL)	3.1	0–23.2
RBC (urine) (cells/µL)	3.3	0–20.8

**Figure 2:**
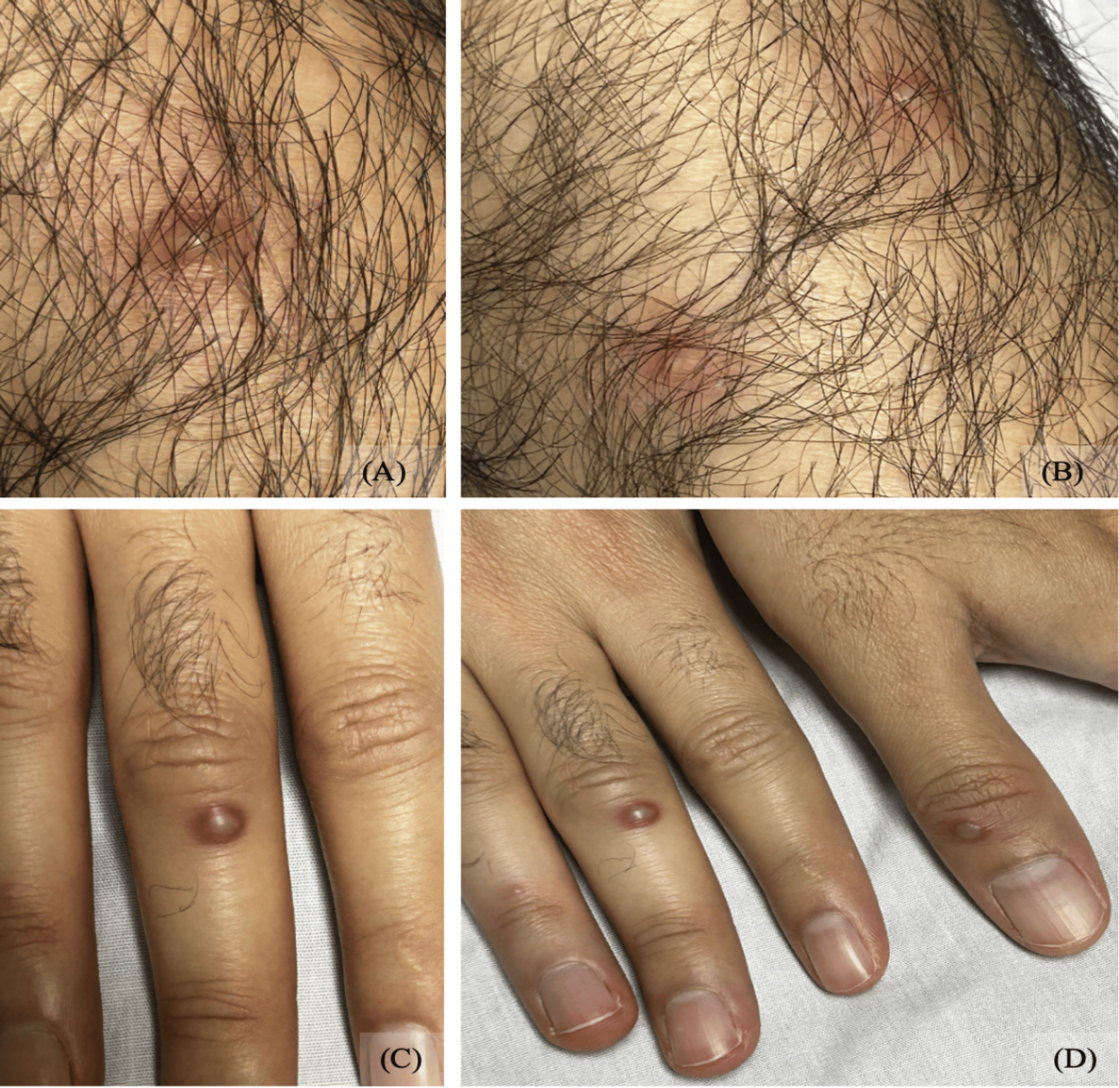
Multiple pustular lesions observed across body regions at day 3: (A-B) erythematous pustules on the right anterior brachium; (C) a pustule over the middle phalanx of the third digit of the left hand; (D) same lesion located over the middle phalanx of the third digit of the left hand, along with an additional pustule on the distal phalanx of the fifth digit of the right hand.

**Figure 3:**
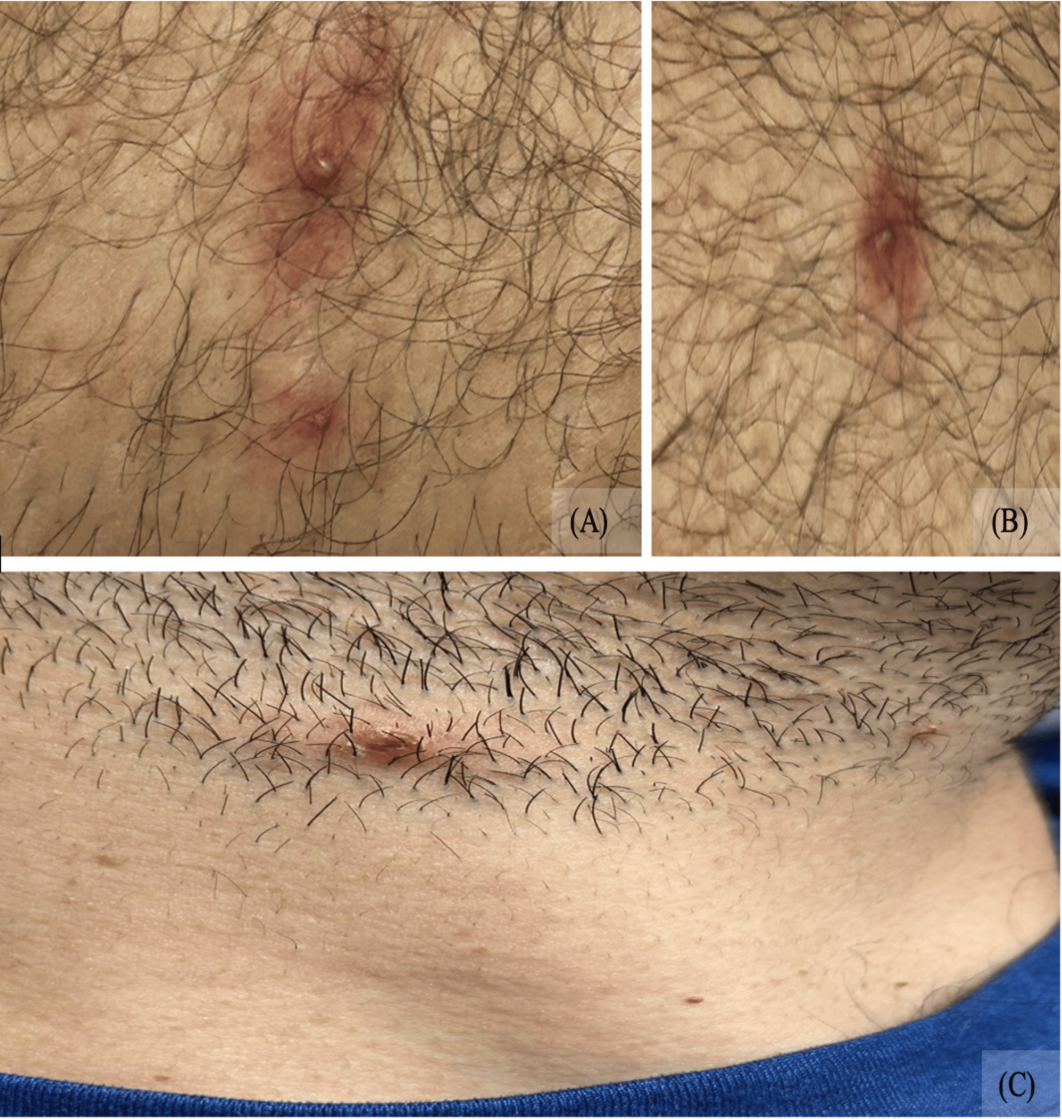
Multiple lesions observed across body regions at day 3: Pustular lesions on the medial right thigh (A–B) and a scabbed lesion on the lateral aspect of the neck (C).

On admission, the pustular lesions had begun to evolve but remained distributed across the same regions noted in the emergency department (Figures 2, 3). Perianal erythema and tenderness were again present, and bilateral inguinal lymphadenopathy was palpable. Upon further discussion, the patient disclosed that he had recently traveled to Minnesota, where he engaged in unprotected receptive anal and oral intercourse with a male partner approximately one week before the onset of symptoms. He denied genital ulcers, urethral discharge, or oral lesions. 

On hospital day 3, Infectious Disease was consulted. Metronidazole was continued, and vancomycin 1,000 mg IV every 12 hours and ceftriaxone 2,000 mg IV daily were added. Ceftriaxone was added to broaden coverage for Gram-negative organisms, including gonorrhea, which can present with proctitis and had not been definitively excluded at that time. Vancomycin was also started to cover possible Gram-positive pathogens, including MRSA, as part of the local approach when symptoms continue and the exact cause has not been identified. Acyclovir 400 mg orally three times daily was also started, given the patient’s history of HSV and the possibility of viral reactivation contributing to anorectal pain. Topical chlorhexidine was applied to reduce the risk of secondary bacterial infection. 

As part of the diagnostic evaluation, serologic testing for HIV and syphilis was performed and returned nonreactive, and HIV-1 RNA PCR was undetectable; nucleic acid amplification testing for gonorrhea and chlamydia was ordered but could not be completed. When the patient began developing umbilicated pustules, Mpox became a primary diagnostic consideration, as Mpox cannot be detected by routine STI testing. A PCR swab was obtained from one of the patient’s hand lesions. STI screening revealed negative results for HIV and syphilis (Table 2). The result was positive for non-variola Orthopoxvirus, confirming Mpox infection. After confirmation and clinical improvement, antibacterial therapy was discontinued. The patient’s hospital course, including key diagnostic milestones and treatment modifications throughout hospitalization, is summarized in Table 3.

**Table 2:** Serologic and infectious testing

Laboratory Test	Patient Value	Reference Range
Serologic & Infectious Testing		
Syphilis (RPR/VDRL)	Nonreactive	Nonreactive
Hepatitis A IgM	Nonreactive	Nonreactive
Hepatitis B surface antigen	Nonreactive	Nonreactive
Hepatitis B core IgM	Nonreactive	Nonreactive
Hepatitis C antibody	Nonreactive	Nonreactive
HIV-1/2 Ag/Ab (4th gen)	Nonreactive	Nonreactive
Influenza A/B	Negative	Negative
SARS-CoV-2 IgM/IgG (rapid)	Negative	Negative
Orthopoxvirus PCR		
Non-variola Orthopoxvirus PCR	Positive	Negative

**Table 3:** Chronological treatment timeline

Hospital Day	Clinical Events & Findings	Medications & Changes
Day 0 (Admission)	Presented with rectal pain, perianal erythema, and new pustular lesions. CT imaging showed findings consistent with proctitis.	Started ciprofloxacin IV and metronidazole IV for presumed bacterial proctitis. Ciprofloxacin was discontinued.
Day 1–2	Persistent anorectal discomfort and evolving skin lesions without significant improvement.	Continued metronidazole IV.
Day 3	Infectious Disease consultation obtained. New pustules and persistent proctitis symptoms were noted. Lesion swab collected for orthopoxvirus PCR.	Ceftriaxone IV and Vancomycin IV were started. Acyclovir PO was initiated due to the patient’s HSV history and ongoing anorectal pain.
Day 4	Clinically stable	Continued vancomycin, ceftriaxone, metronidazole, and acyclovir.
Day 5	Orthopoxvirus PCR positive.	All antibacterial therapy discontinued. Acyclovir continued to complete a five-day course, and topical hydrocortisone was started. The patient discharged the same day with instructions for home isolation and wound care.

We considered several other diagnoses during his evaluation. HSV proctitis was a possibility given his rectal pain, but the lesions were neither vesicular nor ulcerated, and HSV serologic testing was ordered but not completed. Syphilis and lymphogranuloma venereum were also evaluated; syphilis serologies were nonreactive, and no genital ulcers were present. Gonorrhea proctitis was considered less likely based on the absence of mucopurulent discharge and lack of supportive clinical findings, as nucleic acid amplification testing could not be completed. Although bacterial proctitis was initially suspected based on imaging findings, the patient showed minimal clinical improvement despite broad-spectrum antibiotics. The combination of evolving umbilicated pustules, inguinal lymphadenopathy, and a positive lesion-based Orthopoxvirus PCR ultimately supported Mpox as the correct diagnosis.

On October 15, 2025, he was discharged with instructions to continue acyclovir 400 mg twice daily to complete a total five-day course that had been initiated during hospitalization on October 13, 2025. Following confirmation of Mpox and clinical improvement, all antibacterial therapy was discontinued, as no bacterial etiology was identified. He was also prescribed topical hydrocortisone for residual proctitis-related inflammation and instructed to maintain isolation until all lesions had fully crusted and re-epithelialized. Counseling included risk-reduction on high-risk sexual behaviors, the importance of abstaining from sexual activity during the recovery period, and the recommendation to seek evaluation and follow-up at an STD/STI clinic if abstinence could not be maintained to ensure regular screening and prevent potential superinfection. Vaccination options to reduce the risk of future Mpox virus infection were also discussed. Structured outpatient follow-up was not possible, and symptom monitoring was handled exclusively through the Puerto Rico Department of Health; this represents a limitation of this report.

## Discussion

This case demonstrates the course of Mpox in a 32-year-old Puerto Rican male patient with recent high-risk sexual exposure. Although Mpox can be contracted by anyone, regardless of sexuality, it is transmitted through close contact, skin-to-skin contact, exposure to bodily fluids, or fomites. The virus gained widespread clinical attention following the 2022 outbreak, during which cases increased among men who have sex with men.

The typical incubation period ranges from 7 to 10 days, allowing patients to unknowingly transmit the virus before symptoms appear. During the 2022 outbreak, many individuals experienced atypical or mild presentations and did not initially associate their symptoms with Mpox. Some developed limited anogenital lesions with few or no systemic symptoms [1,3,7]. Mitjà and colleagues observed that men who engage in anal-receptive intercourse were more likely to present with proctitis [1], which aligns with our patient’s CT findings and symptom profile (Figure 1). Because localized lesions, rectal discomfort, and mild systemic symptoms closely resemble conditions such as HSV, syphilis, gonorrhea, or LGV, Mpox is often misdiagnosed in low-incidence regions, which may contribute to diagnostic delays, explaining why our patient was initially treated for bacterial proctitis.

In atypical cases, Mpox may involve only a few lesions with limited distribution. When present alongside anorectal pain and mild systemic involvement, these findings can easily resemble common sexually transmitted infections, making careful evaluation essential [5]. Lesion morphology (such as umbilication), the sequence of lesion evolution, and inguinal lymphadenopathy can provide valuable diagnostic clues when Mpox is not immediately suspected.

Between 2022 and May 2025, a total of 267 Mpox cases were confirmed in Puerto Rico, most of them in men. A substantial portion of cases occurred in individuals between 30 and 39 years old, matching the pattern reported in other parts of the world. Nearly three-quarters of patients said they had sexual activity within 21 days before their symptoms began, supporting evidence that close, intimate contact remains the main route of transmission. About 15.8% of confirmed cases had recently traveled, suggesting that both local spread and imported infections have contributed to the reported cases [8]. Delayed suspicion is common in regions with low case numbers, and persistent underreporting limits clinicians’ familiarity with atypical presentations.

Although the incidence has declined since 2022, maintaining clinical awareness remains essential. Persistent underreporting in low-incidence regions contributes to delayed Mpox detection and response. Making sure clinicians stay current through routine education, refreshed protocols, and practical training can strengthen awareness and support earlier recognition and reporting of unfamiliar or imported cases. These gaps in reporting and familiarity likely contributed to the initial attribution of this patient’s symptoms to bacterial proctitis, delaying appropriate suspicion for Mpox.

When compared with cases reported in the United States and Latin America, this patient’s presentation was very similar to what has been described in Mpox infections linked to sexual contact. Between May and July 2022, Philpott et al. reported 2,891 confirmed cases in the United States, with 94% reporting recent male-to-male sexual activity and 99% in men [9]. The majority of patients had vaginal or anal lesions with relatively mild systemic symptoms, according to the analysis of 528 cases from 16 countries by Thornhill et al. [10]. The resemblance between our patient's presentation and the patterns observed in these studies emphasizes how crucial it is to remain vigilant about sexually transmitted Mpox, particularly in areas where a low number of cases may reduce clinical suspicion. This comparison highlights how lesions with limited distribution, mild systemic involvement, and prominent anorectal symptoms can closely resemble more common STIs, making Mpox an important consideration in similar clinical settings.

Mpox was historically confined mostly to Central and West Africa, but in the last few years, its spread has expanded to other countries due to international travel and ongoing person-to-person transmission [2,3]. This case highlights the importance of recognizing sexually transmitted infections that can present atypically, especially in regions like Puerto Rico, where such infections are uncommon. A thorough, judgment-free sexual history is vital to accurate diagnosis and timely treatment. Creating a welcoming, non-judgmental environment encourages patients to disclose sensitive details that may otherwise be withheld due to stigma or fear of prejudice [11,12]. Lessons from the early HIV epidemic illustrate how bias and misinformation can delay diagnosis and erode trust between patients and clinicians [13]. Applying these lessons today, particularly with emerging infections such as Mpox, underscores the need for inclusive, empathetic communication to promote early detection, equitable care, and better public health outcomes. This case reinforces that even in a low-incidence environment, clinicians must keep Mpox in their differential diagnosis when faced with STI-like lesions or unexplained proctitis, especially in sexually active patients with recent travel or new partners.

## Conclusions

This case demonstrates that Mpox may present primarily with proctitis and a few STI-like lesions. The patient’s localized umbilicated pustules, limited lesion burden, and absence of early systemic symptoms contributed to the initial assumption of bacterial proctitis and delayed consideration of Mpox. This issue can be especially challenging in low-incidence regions such as Puerto Rico. Linking these atypical features with the patient’s recent travel and high-risk sexual exposure highlights the importance of maintaining a broad differential diagnosis. Recognizing lesion morphology, timing, and associated anorectal symptoms can help clinicians distinguish Mpox from conditions like HSV, syphilis, or gonorrhea and prompt earlier testing. Early recognition, appropriate isolation, and timely communication with public health authorities remain critical to preventing further transmission. This case emphasizes the need for thorough assessment, inclusive sexual history taking, and clinician education to prevent missed Mpox diagnoses.
